# Practical Notes and Formulæ

**Published:** 1887-11

**Authors:** 


### PRACTICAL NOTES AND FORMULÆ.

**Soothing Mixture for Consumptives.**—Of all the mixtures for stimulating the consumptive patient, allaying his cough and quieting his nervousness, I have found no equal to the following:

℞. Syrup liquorice............................℥ j,

Aromatic syrup rhubarb.........................℥ ss,

Fluid extract of opium.........................ʒ i,

Liquor ammon. acetat...........................℥ v.

M. Sig.—Shake well. Dose, a tablespoonful every two or three hours.

Patients become very fond of this mixture, and it in no wise interferes with the stomach or appetite. Should constipation ensue, it is easily overcome by an occasional dose of com. liquorice powder.—*Medical and Surgical Reporter.*

**Barium Chloride in Vascular Diseases.**—Kobert, of Dorpat, Russia, writes as follows in the *Therapeutic Gazette* of June 15, 1887: The best application for dilated cutaneous veins (for example, on the legs) is the following:

℞. Barii chloridi..............................gr xxx,
Dissolved in distilled water and mix thoroughly.
Lanolini.......................................ʒ 3¾,
Olei amygdalarum dulc..........................lxxv *m.*

M. F. Unguentum. Sig.—Three times daily, with friction, whenever diluted blue viens shine through the skin.

Barium chloride can also be used hypodermically in place of substances of the digitalis group, in cases of heart disease where help is demanded so soon that remedies given internally are not available.—*Peoria Medical Journal.*

**Pruritus of the Female Genitals.**—The following formula is recommended by Meigs for pruritus vulvæ:

℞. Boracis.....................................ʒ vi

Morphinæ hydrochlor............................gr. vi,

Aquæ roses.....................................ʒ viss.

M. Sig.—Bathe the parts affected.

Between the applications, lycopodium or starch flower may be dusted upon the affected parts.

**Treatment of Hepatic Conjestion.**—Jules Cyr gives (Rev. de Terap.) the following rules for the treatment of the above: 1. Application over the liver of compresses of cold water, often renewed; two or three leeches about the anus. 2. At evening, ¾ of a grain of calomel should be taken, followed the next morning by five drachms of Glauber's salts. 3. As beverage, milk and Vichy water, or 75 grains of ammonium chloride in a quart of water. A douche, while the patient is reclining, of water at a pleasant temperature, given over the hepatic region.—*Medical Age.*

**Syrup Trifolium Comp. in Syphilis.**—Last spring a young man came to me to be treated for syphilis, in the secondary stage. He stated he had been suffering from it for several years, and had undergone the usual mercurial treatment, and to use his expression, "the Lord knows what all." He was thin, nervous, had sores on both shins, and, in fact, was "all broke up." His lower lip was very much swollen and scarred from the effects of mercury. He was also suffering from what he thought was asthma ; but this was only from malnutrition. I decided to try Syrup Trifolium Comp., which I gave in combination with 5 grs. citrate of iron three times a day, for about two weeks. He began to improve right away. The asthma, swollen lip, and sores disappeared and he gained rapidly in flesh. He is still taking a teaspoonful of the syrup two or three times a day, and is apparently a sound man.—DR. M. B. KETCHUM, *in Medical Age.*

**An Enema for Convulsions of Children.**—*Nouveaux Remedes* attributes the following formula to Dr. J. Simon :

| | |
|---|---|
| Musk | grs. iij, |
| Camphor | grs. xv, |
| Chloral hydrate | grs. vij, |
| The yolk of one egg | |
| Distilled water | ℥ iv. |

The rectum is to be emptied by a simple enema before this is injected.—*N. Y. Medical Journal.*

**Treatment of Flatulent Dyspepsia.**—Huchard recommends the following formulæ :

| | |
|---|---|
| ℞. Salicylate of bismuth | parts ij, |
| Calcined magnesia | " ji, |
| Powdered willow charcoal | " iii, |
| Oil of anise | " i. |

Of this powder a small teaspoonful may be taken an hour or a half-hour before a meal. When gastralgia is added to flatulent dyspepsia, he recommends the following :

| | |
|---|---|
| ℞. Syrup of peppermint | parts ccl, |
| Hydrochloric acid | parts i, |
| Hydrochlorate of cocaine | parts 1-10. |

of which a small glass (such as those in which liqueurs are served) may be taken after a meal.—*Revue Generale, de Clinique et de Therapeutique*, May 19, 1887.

**For Mammary Inflammation.**—

| | |
|---|---|
| ℞. Tinct. aconiti, rad | gtt. x, |
| Tinct. phytolaccæ decan | gtt. xx, |
| Aquæ | ℥iv. |

M. Sig.—One teaspoonful every hour, and have applied to the gland equal parts of tinct. phytolacca and water, every three hours."—*Medical Summary.*

